# Shea (*Vitellaria paradoxa* Gaertn C. F.) fruit yield assessment and management by farm households in the Atacora district of Benin

**DOI:** 10.1371/journal.pone.0190234

**Published:** 2018-01-18

**Authors:** Koutchoukalo Aleza, Grace B. Villamor, Benjamin Kofi Nyarko, Kperkouma Wala, Koffi Akpagana

**Affiliations:** 1 West African Science Service Centre for Climate Change and Adapted Land Use (WASCAL) Graduate Research Programme, School of Agriculture, Department of Soil Science, University of Cape Coast, Ghana; 2 Laboratoire de Botanique et Ecologie Végétale, Université de Lomé, Lomé, Togo; 3 Centre for Development Research, University of Bonn, Germany, Bonn, Germany; 4 Department of Geography and Regional Planning, University of Cape Coast, Cape Coast, Ghana; Chinese Academy of Forestry, CHINA

## Abstract

*Vitellaria paradoxa* (Gaertn C. F.), or shea tree, remains one of the most valuable trees for farmers in the Atacora district of northern Benin, where rural communities depend on shea products for both food and income. To optimize productivity and management of shea agroforestry systems, or "parklands," accurate and up-to-date data are needed. For this purpose, we monitored120 fruiting shea trees for two years under three land-use scenarios and different soil groups in Atacora, coupled with a farm household survey to elicit information on decision making and management practices. To examine the local pattern of shea tree productivity and relationships between morphological factors and yields, we used a randomized branch sampling method and applied a regression analysis to build a shea yield model based on dendrometric, soil and land-use variables. We also compared potential shea yields based on farm household socio-economic characteristics and management practices derived from the survey data. Soil and land-use variables were the most important determinants of shea fruit yield. In terms of land use, shea trees growing on farmland plots exhibited the highest yields (i.e., fruit quantity and mass) while trees growing on Lixisols performed better than those of the other soil group. Contrary to our expectations, dendrometric parameters had weak relationships with fruit yield regardless of land-use and soil group. There is an inter-annual variability in fruit yield in both soil groups and land-use type. In addition to observed inter-annual yield variability, there was a high degree of variability in production among individual shea trees. Furthermore, household socioeconomic characteristics such as road accessibility, landholding size, and gross annual income influence shea fruit yield. The use of fallow areas is an important land management practice in the study area that influences both conservation and shea yield.

## 1. Introduction

*Vitellaria paradoxa* (Gaertn C. F.), commonly known as shea tree produces an edible fruit that is the source of one of Africa’s most ancient food oils. Shea trees are indigenous to semi-arid and sub-humid savannas of sub-Saharan Africa (SSA), occurring on nearly 1 million km^2^among 18 African countries[1, 2]. Currently, shea butter, the main product of this tree, is sold on local, domestic and international markets for baking, confectionery, cosmetic and pharmaceutical purposes. In contrast to other important regional cash crops such as cotton and cashew, shea tree production benefit from few integrated development efforts that represent meaningful investments in improving management practices; furthermore, involvement in the shea industry has been concentrated almost exclusively on improving processing and marketing. As a result, despite seven centuries of commercial shea butter trade in areas beyond its geographic distribution, it essentially remains a semi-domesticated resource[3].

Shea agroforestry parklands, also known as “shea parklands” in short, have received international attention since the 1950s, when shea tree products became recognized as important nutritional and economic resources. Early studies characterized shea parklands as an indigenous farming system [4–6]. Several studies have investigated the extent of these stable and integrated systems, their role in local economies and related stakeholders, and described tree resource stocks and demographic structure of shea tree population in the area where shea tree occurs[7–11]. Furthermore, shea parklands located in arid and semi-arid areas are considered an important agro-ecosystem for carbon sequestration and maintaining soil conditions [12, 13]. In addition, model simulations using Intergovernmental Panel on Climate Change (IPCC) scenarios predicted that shea distribution in 2020, 2050 and 2080 might not be affected by climate variability on a regional scale [14]. This suggests that shea parklands could be a resilient land use with respect to the effects of climate change in West Africa. In terms of livelihoods, shea butter was the main edible oil for more than 80% of the rural population of northern Benin about 30 years back [5] and third most important export crop in the country[15]. Shea trees are a potential farm crop option for poverty reduction efforts in the region. Apart from environmental benefits, shea tree products provide at least 35% of the annual income to rural communities, especially women, in the Atacora and Donga districts of northern Benin [16].

However, the shea nut, from which most shea products are extracted, has an unpredictable and complex production pattern. The species’ slow growth rate, extended juvenile life stage, genetic variability and complexity of interactions between shea trees and other components of local ecosystems are among the factors that limit shea productivity. Research efforts have addressed issues of reproduction and management through tree nurseries [17], but the dissemination of such techniques is still lacking. In view of the improvements to the productivity and quality of shea tree resources, grafting trials were performed and showed potential to enhance the proliferation of desirable genotypes and reduce the length of the juvenile life stage[11, 18], juvenile life stage that varies between 15 and 20 years under natural conditions. Fruit production typically commences at 20 years of age, but production is not maximized until 40 to 50 years of age[17]. Relatively little is known about factors that govern productivity. Fruit production is described as cyclic by some authors [19, 20], whereas others attribute yield variations to the genetic variability of individual trees[4, 17, 21]. Soro et al.[22] showed that shea productivity in Cote d’Ivoire increases with mean monthly rainfall. That study was conducted over five consecutive years and the findings suggested that shea productivity might be linked to rainfall and less directly to temperature. In Benin, Glèlè et al. [23] found an increase in shea production from the Sudano-Guinean to the Sudanian zone, whereas little is known about the linkages between shea production and land-use types [24]. In this study, the overall objective was to establish an understanding of shea productivity in relation to biophysical variables, soil groups, and management practices. Specifically, this study addressed the following research questions:

1. What is the current shea fruit yield pattern in Atacora?
2. What physical and morphological factors contribute to shea yields, and are these factors good predictors of fruit yield?
3. What factors affect farm household livelihoods in the study area that may also affect conservation of shea parklands?

## 2. Material and methods

### 2.1. Study area

**Fig 1:**
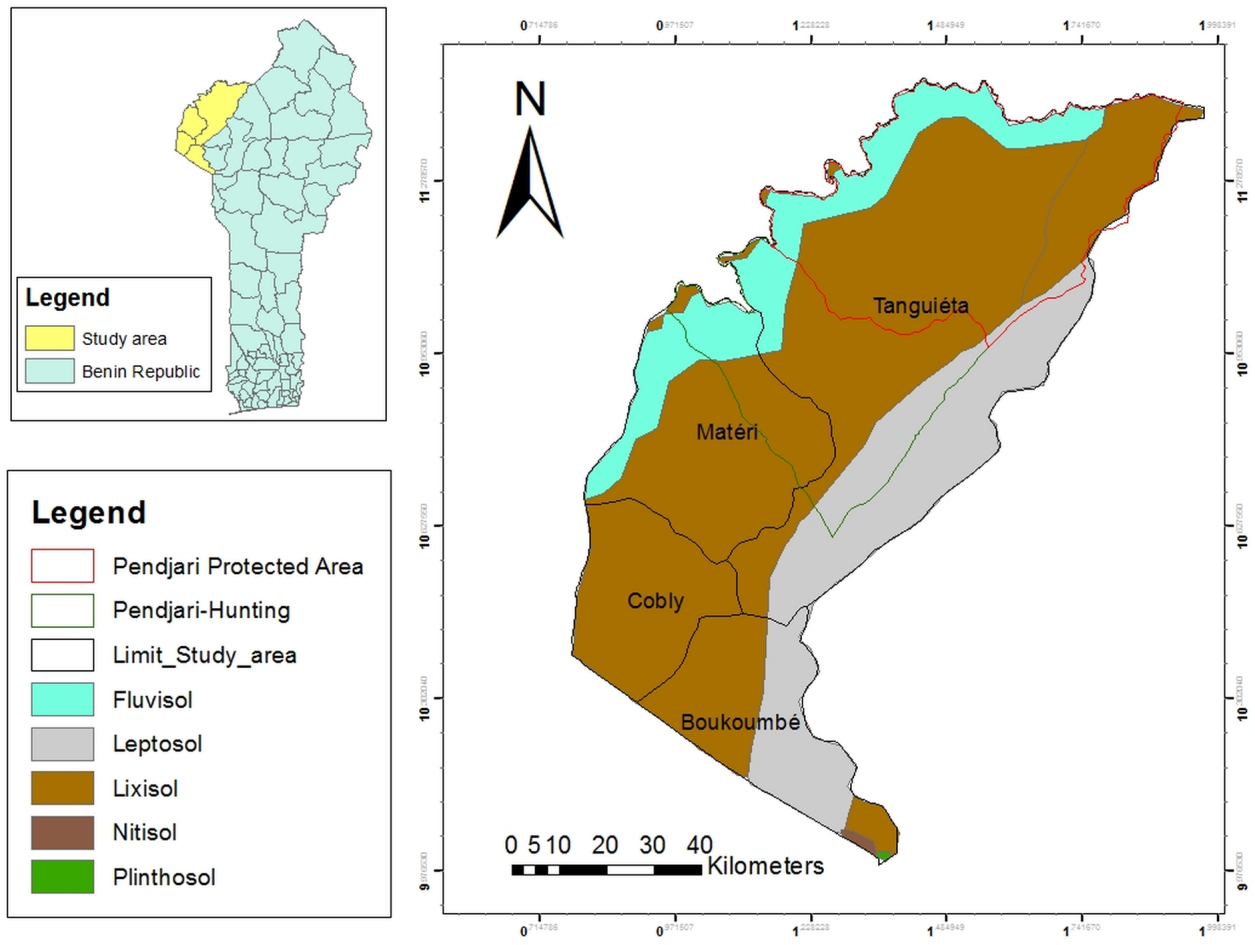
Soil group of the study area in the Atacora district in northern Benin.

This study was conducted in two communes (i.e., Tanguieta and Materi) in Atacora located between 10°and 12° north, and 0°and 2°east, in the Sudan zone of northern Benin (Fig 1). According to INSAE [25], the district has a population of 769,337 (representing approximately 7.71% of the country’s total population), of which 70% is rural and dependent on agricultural production as their primary livelihood means. A mountain range known as the ‘Atacora chain’ extends along the northwest border of the district and extends into northeast Togo. The average annual temperature in the district is 27°C and temperatures range from 17°C to 35°C. The climate in Atacora is characterized by two seasons: a rainy season from April to October that exhibits intra-annual variability and a relatively dry season the rest of the year. Mean annual precipitation is 1,271 mm. Vegetation at the study site is dominated by woodlands and riparian forests along the Pendjari river and its tributaries. Common tree species include *Pterocarpuserinaceus* (Poir.), *Lophiralanceolata* (Tiegh. ex. Keay), *Anogeissusleiocarpa* (DC, Guill. And Perr.), *Isoberliniadoka* (Craib. And Stapf.) and *Khaya senegalensis* (Desr. A. Juss.). Numerous varieties of annual crops such as cotton, maize, sorghum, groundnut, cowpea, millet, etc., are cultivated in association with scattered multi-purpose trees such as *V*. *paradoxa* and *Parkiabiglobosa* (Jacq. Dong.).

#### 2.1.1. Land-use types

Two basic land-use types were considered:farmlands and fallows. Farmlands include areas where annual crops are actively cultivated, whereas fallows are areas where annual crops were previously cultivated that have been left to rest in order to restore soil structure and fertility. These are the dominant land management regimes defined by scholars for shea parklands [3, 26–28]. Fallow areas were further divided into two groups according to their age: young, for areas that have been left fallow from one to five years, and old for areas left fallow for a period above five years. The common cycle known in the Soudan zone is one to five years for short fallows and more than five for long ones[29, 30] with some reaching 20 years or more. Regardless of the land-use type, shea tree age not considered. The separation of fallow areas by age class was intended to explore the implication of fallow age to shea parklands management and fruit yield. During visits to particular plots, the distinction between young and old fallows was based on the presence of *Andropogongayanus* (Kunth) a species indicative of good soil fertility[31], high woody species density and diversity[32]. In ambiguous cases it was necessary to directly inquire about fallow age with the plot owner.

#### 2.1.2. Soil groups

According to the FAO soil classification, three soil groups (Leptosols, Lixisols and Fluvisols) are the most widely represented in the study area. Their descriptions provided below are from Jones et al.[33]. Leptosols are shallow soils over hard bedrock that contain a relatively high proportion of gravel or highly calcareous deposits and account for approximately 17% of the land area of Africa. Leptosols have a limited pedogenic development, which contributes to weak soil structure. This group of soil occurs all over the African continent, especially in mountainous and desert regions where hard bed rock is often exposed or close to the surface. In northern Benin, Leptosols are found along the Atacora Mountains. The physical disintegration of rock due to heating and cooling cycles is the main soil formation process. Leptosols are not considered arable and have limited potential for tree crop production or livestock grazing. On Leptosols trees must be shallow rooted and develop best in areas with deeper soil and where poor drainage improves moisture retention. Leptosols are most productive when forested. Lixisols are slightly acidic soils that exhibit a distinct increase in clay content with depth. The predominance of kaolinite limits the capacity of this soil group to retain nutrients. Supporting savannahs or open vegetation with low biomass production, Lixisols do not retain much organic matter and lack a well-developed soil structure. These soils are characterized by low levels of plant nutrients and high erodibility, which make agriculture possible only with frequent fertilizer applications, minimum tillage, and erosion control. Perennial crops are more suitable for this soil group than root or tuber crops. Fluvisols occur in swampy areas drained by the Pendjari River and its tributaries and are inappropriate for shea tree establishment.

### Research methods

#### 2.2.1 Dendrometric parameters measurements and requirement

For each fruiting shea tree selected we collected data on certain dendrometric parameters such as diameter at breast height (DBH) or 1.3 m, crown height, crown diameter(diameter north-south and east-west), total height and the height at first branching. We recorded the coordinates of selected shea trees using GPS devices for mapping and reference purposes.

Shea tree, the species under study, is a protected species and for that matter a formal permission from national authorities was needed prior to the field work. Permission was given by the Ministry in charge of forest and natural resources in Cotonou (Benin). For each of the investigated villages, an oral permission was given from the head of the village or the district chief. No other animal was involved in this study.

#### 2.2.2 Fruit yields

**Fig 2:**
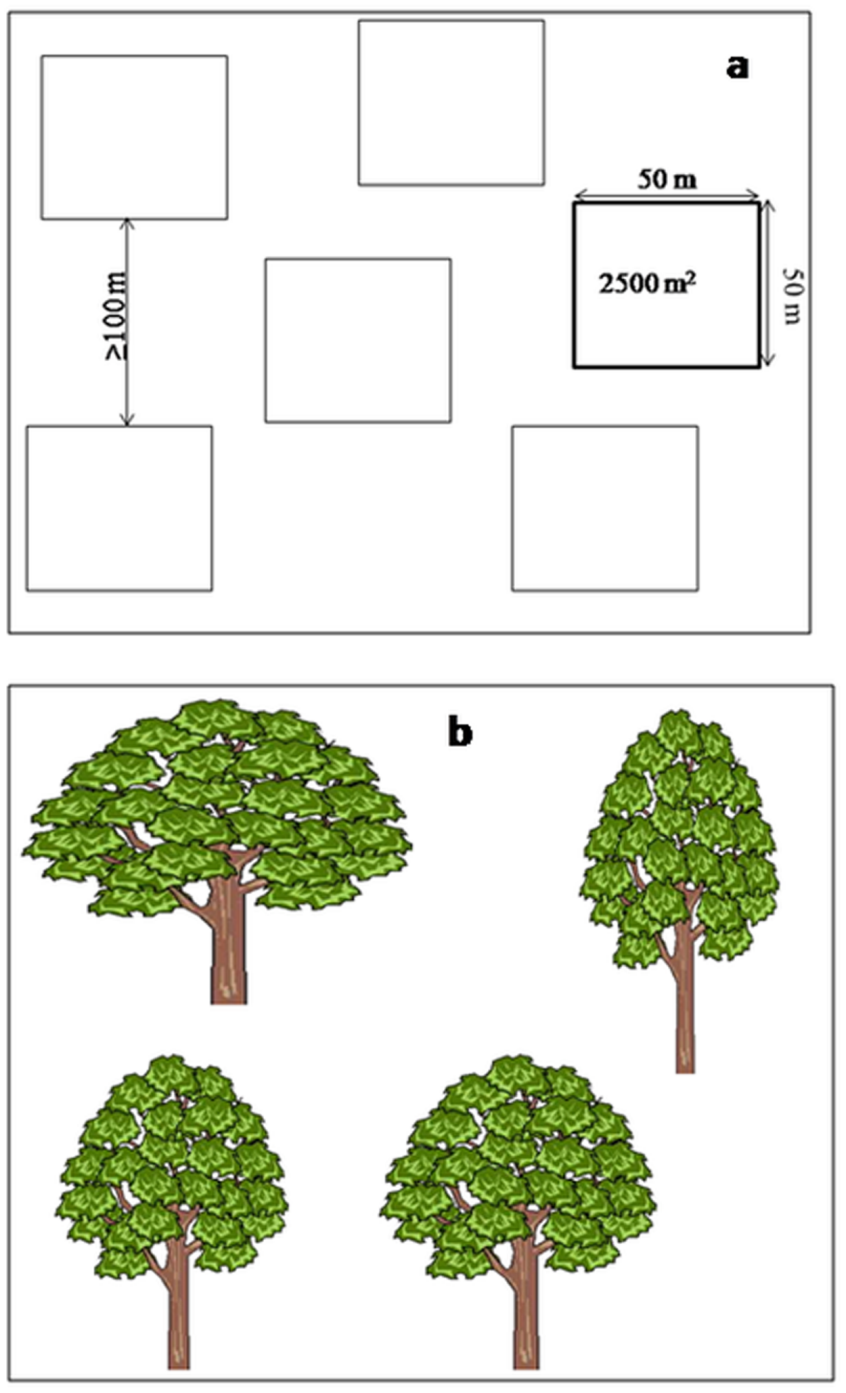
Sampling design for (a) shea tree productivity assessment for each land use type, based on (b) four fruiting trees per plot.

Annual shea tree fruit yields were assessed directly by measuring fruit production. Further, the potential fruit yield was derived from farming household characteristics reported in the survey based on the measured yield. Based on the soil groups of the research site, we selected six villages within the Tanguieta and Materi communes. Since Leptosols and Lixisols were well represented in the two communes, shea fruit yield was measured only in the two communes. On each major soil group, shea fruit yield was assessed on farmland, young fallow, and old fallow sites[34]. In both Leptosols and Lixisols, six (06) plots (50 m × 50 m) were established in farmland, six (06) in young fallow and three (03) in old fallows. Within each plot four fruiting shea trees were randomly selected for yield evaluation (Fig 2). A total of 60 fruiting shea trees (*n* = 24 for farmland, *n* = 24 for young fallow and *n* = 12 for old fallow) were monitored on each of the two soil groups for two consecutive growing seasons: from 2013 until 2015.

Although we originally intended to sample each land use equitably, old fallow sites are relatively scarce in the study area and most of the trees found in old fallow areas were not productive. Hence the total sample of trees on old fallow plots was reduced from 48 to 24.

Fruit yield per tree was measured using the randomized branch sampling method [35, 36]. This approach consists of sampling secondary branches from main branches off the primary stem. For each tree, the total number of main branches was recorded and four were selected from the total. Four secondary branches along each main branch were randomly selected for sampling. The number of fruit along each selected secondary branch up to the terminal segment was counted. The number of fruit for each of the four selected branches was the product of the number of fruit observed along each counted stem and the number of forks along the path of the main branch. Pooled fruit yield was estimated from the mean number of fruits on the selected branches of each tree. The most commercially important shea tree product is the nut. To estimate the parameters of the nut yield, a total of 10 mature fruit was randomly selected from each fruiting tree and weighed. Afterwards, the fresh pulp was removed from each nut for a second weighing. The nuts were then sun dried and weighed again.

#### 2.2.3 Farm household survey

A multistage sampling design was used to select households to be interviewed. Four communes in Atacora District were selected; Boukombé, Cobly, Materi and Tanguieta. In each commune representative localities were randomly chosen and households selected based on their implication in shea parklands management. A mixed methods approach that combines household survey and field observations was adopted. A total of 200 farm households were surveyed using semi-structured questionnaires requesting socio-economic and farm characteristics of each household as well as management practices for conserving shea parklands and factors affecting their livelihoods. From the households socio-economic characteristics and management practices recorded in the survey was derived the potential fruit yield for each of the 200 households.

### 2.2. Data analysis

Dendrometric parameters (diameter and total height) from the three land-use types were compared using a one-way analysis of variance (ANOVA).

To compute annual fruit yields of individual trees we used a Microsoft Excel spreadsheet with the following equation:

$$P\;=\;\frac{F}{4}\sum_{n\;=\;1}^{4}\left[\frac{ni}{4}(\sum_{i\;=\;0}^{4}Pi)\right]$$ (1)

where F is the total number of main branches on a selected tree, *ni* the number of secondary axes along a sampled main branch, and P*i* is the number of fruit counted along secondary stems off the main branch.

We examined the relationships between dendrometric parameters, and soil and land-use types and shea fruit yields. Fruit mass was used for yield prediction models. The dataset on shea fruit yield failed to satisfy assumptions such as normality and uncorrelated residuals necessary for linear regression. There were number of outliers (which happen to be real data) that had an influence on the analysis. A regression model (with Breusch-Pagan / Cook-Weisberg test for heteroskedasticity) with robust standardized errors was used to cut off the aforementioned issues. Yield prediction models were established based on regression analysis results using Stata 13 software. We also checked for multicollinearity using a variance inflation factor (VIF) analysis. An alpha level of 0.05 was used for all statistical tests.

We estimate shea fruit yield per household through the potential fruit yield and assessed socio-economic factors and management practices that might be associated with it. The potential fruit yield of each farm household (hereafter potential fruit yield) is function of the total area of land managed for shea production (including each of the three land-use types) by the household, shea tree density (by land-use type) based on previous studies by the authors [34], and the geographical location. Tree density was averaged by location, since it varies by and is generally uniform within communes (see Eq 2). The gross income of farm households was derived from the 2013 crop yield and their corresponding prices on local market prices. An average of post harvest and off-season prices was applied to calculate gross income. A regression analysis was applied using Stata13 software to identify the factors affecting the potential fruit yield and the adoption of management practices.

$$Y_{h}\;=\;\sum_{i\;=\;1}^{S}\left[S_{i}{D_{i}P}_{i}\right]$$ (2)

where, Pi is the observed fruit yield in land-use type *i*; Di is shea tree density in land-use type *i*; Si is the area of land under land-use type *i*; *i* = 1 for farmland, *i* = 2 for young fallow, and *i* = 3 for old fallow.

## 3. Results

### 3.1. Shea tree dendrometric parameters

Shea trees in farmland had the largest diameters, followed by trees on old fallow and young fallow plots (Table 1). Fisher test results indicated that the mean tree diameter on farmland differed significantly (p-value = 0.021) from on young fallow, but not from mean diameter on old fallow. Mean shea tree height in young fallow was significantly (p-value = 0.003) lower than in farmland and old fallow.

**Table 1:** Shea tree dendrometric parameters and fruit mass according to land-use type.

Land use	Mean diameter (cm)	Mean height (m)
Farmland	35.3 ± 10.5^a^	12.9 ± 3.7^a^
Young fallow	29.9 ± 9.3^b^	10.6 ± 3.9^b^
Old fallow	31.4 ± 8^a^^b^	13.3 ± 4.1^a^

^a^ and ^b^refers to Fisher’s tests at 95% confidence interval; means that do not share letters are significantly different.

### 3.2 Shea tree fruit yield dynamics

#### 3.2.1 Effect of land-use type

Fruit yield varies according to land-use type. Table 2 displays the temporal and spatial variability of fruit yield. Fruit mass in farmland (17.4 kg of fresh fruit per tree) is significantly (p = 0.05) higher than the one registered in young and old fallows. There is nearly 5 and 6 kg difference in fruit mass from farmland to young fallow and from farmland to old fallow respectively “S2 Table”.

**Table 2:** Shea fruit yield according to land-use type.

Land-use type	Fruit mass (kg)	Stdev	Number of fruit			
			Year 1	Stdev	Year 2	Stdev
Farmland	17,4^a^	11,7	1136	955	791	742
Young fallow	12,5^b^	11,9	649	568	715	855
Old fallow	11,4^b^	11,0	620	519	916	1018

Note: Means that do not share a letter are significantly different

Inter-annual variability in shea fruit yield differed by land-use type (Table 2), but the differences were not statistically significant. On farmland, yield dropped from 1,136 fruit per tree in 2013–2014 to 791 in 2014–2015, or nearly 30%. In contrast, yield on young fallow increased by nearly 10% (from 649 to 715 fruit per tree) and yield on old fallow increased by 48% over the same period.

#### 3.2.2 Effect of soil group

Soil group has little effect on fruit yield as compared to land-use type (Table 3). Lixisols registered the highest fruit mass (15.8 kg). Though the latter soil group registered almost 3 kg more than Leptosols, no significant difference was observed in fruit mass. Unlike the case in land-use type, the inter-annual variability in fruit yield was not important. A reduction of about 7% was observed in Lixisols from year 1 to 2 while no change was registered on Leptosols.

**Table 3:** Shea fruit yield according to soil group.

Soil group	Fruit mass (kg)	Stdev	Number of fruit			
			Year 1	Stdev	Year 2	Stdev
Lixisols	15,8^a^	13,5	919	938	814	861
Leptosols	12,7^a^	9,8	757	562	757	833

Note: Mean that do not share a letter are significantly different

#### 3.2.3 Combined effect of land-use type and soil group

A pair-wise comparison of fruit yield based on the combination of soil group and land-use types was done. Average fruit yield in farmland was 4 kg greater on Lixisols than on Leptosols; but the difference was not significant due to the high variability among trees. Meanwhile, there was a statistically significant difference in young fallow fruit yield between Leptosols and Lixisols. Trees in young fallow under Lixisols conditions registered almost the double of the yield registered by those on Leptosols. A similar pattern was observed on old fallow plots on Leptosols where yields were double the mass of yields on Lixisols (Table 4).

**Table 4:** Pair wise comparison of marginal predictions results for mean fruit mass by land use and soil group.

Variable	Margin	Std. Err.	Unadjusted Groups
Combined Land-use and Soils:			
Lixisols#Farmland	19.5	2.3	B
Leptosols#Farmland	15.4	2.3	B
Lixisols#Young fallow	16.4	2.3	B
Leptosols#Young fallow	8.5	2.3	A
Lixisols#Old fallow	7.3	3.2	A
Leptosols#Old fallow	15.6	3.2	AB

Note: Margins sharing a letter in the group label are not significantly different

### 3.2 Shea fruit yield predictive model

#### 3.3.1 Factors associated with shea fruit yield

**Fig 3:**
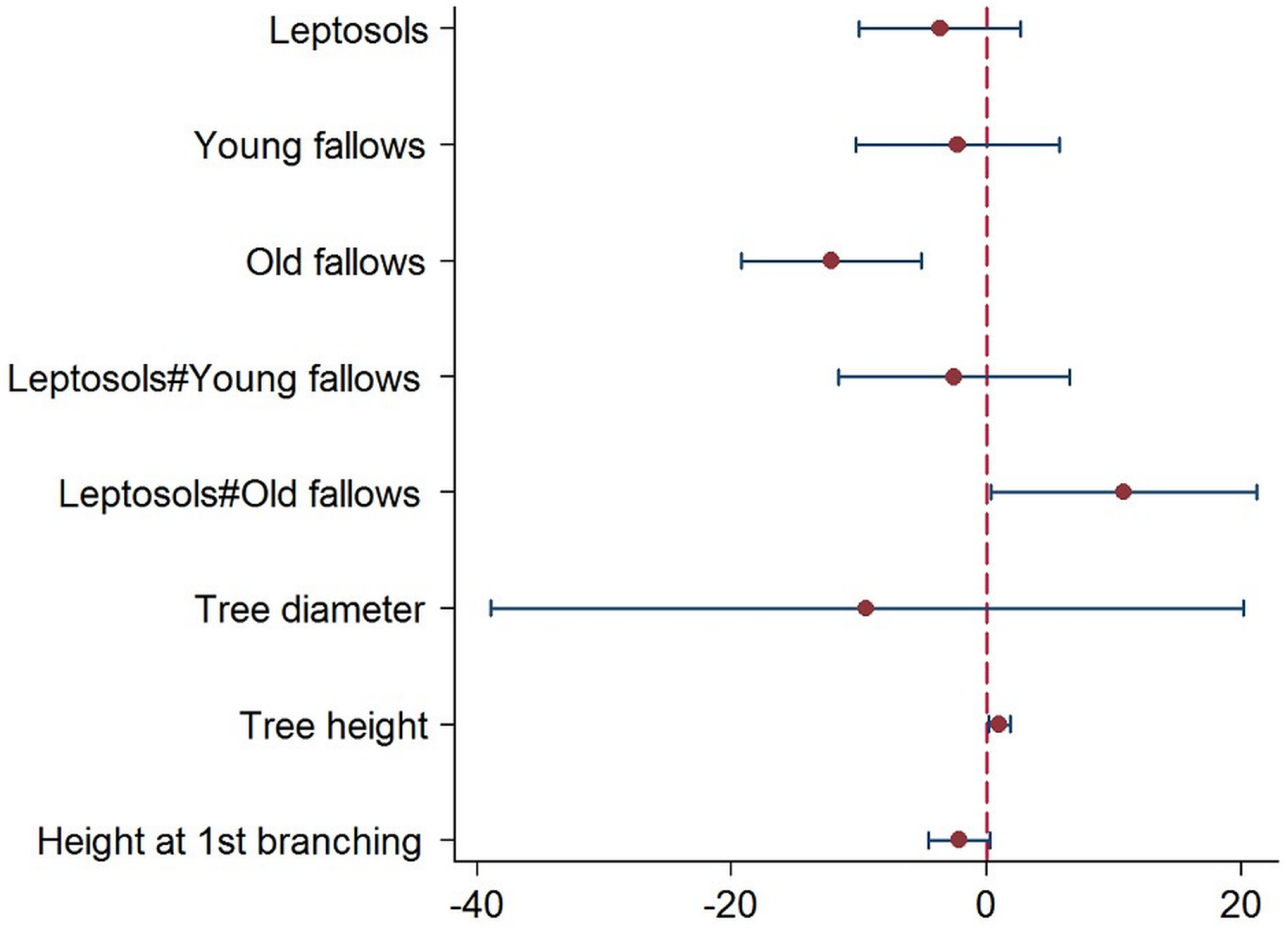
Effects of dendrometric parameters, soil and land-use types on fruit yields. (Note: The test for heteroscedasticity results were significant, p = 0.0001; *n* = 120; Prob> F = 0.0002; R^2^ = 0.2031). Variable (y axis) with negative coefficient (x axis) are note associated with fruit yield.

The shea fruit yield prediction model was established based on dendrometric parameters, soil and land-use types (Fig 3). The probability of the resulting model is significant (p = 0.00) at R^2^ = 0.20, indicating that 20.3% of the observed variation in fruit yield is accounted for by the model. Fruit yields were significantly different in old fallow (p = 0.00) and on old fallow plots on Leptosols (p = 0.04) versus Lixisols. Total shea tree height was positively (b = 1.03) and significantly (p = 0.01) associated with fruit yield.

Although there were no significant effects of Leptosols (p = 0.22), young fallow (p = 0.44), and young fallow on Leptosols (p = 0.42), there was a significant but negative effect of old fallow on fruit yields (p = 0.00). On Leptosols, however, old fallow had a positive relationship with fruit yield. There were no relationships between fruit yields and dendrometric variables such as DBH, tree height, and height at first branching.

#### 3.3.2 Soil group effect

**Fig 4:**
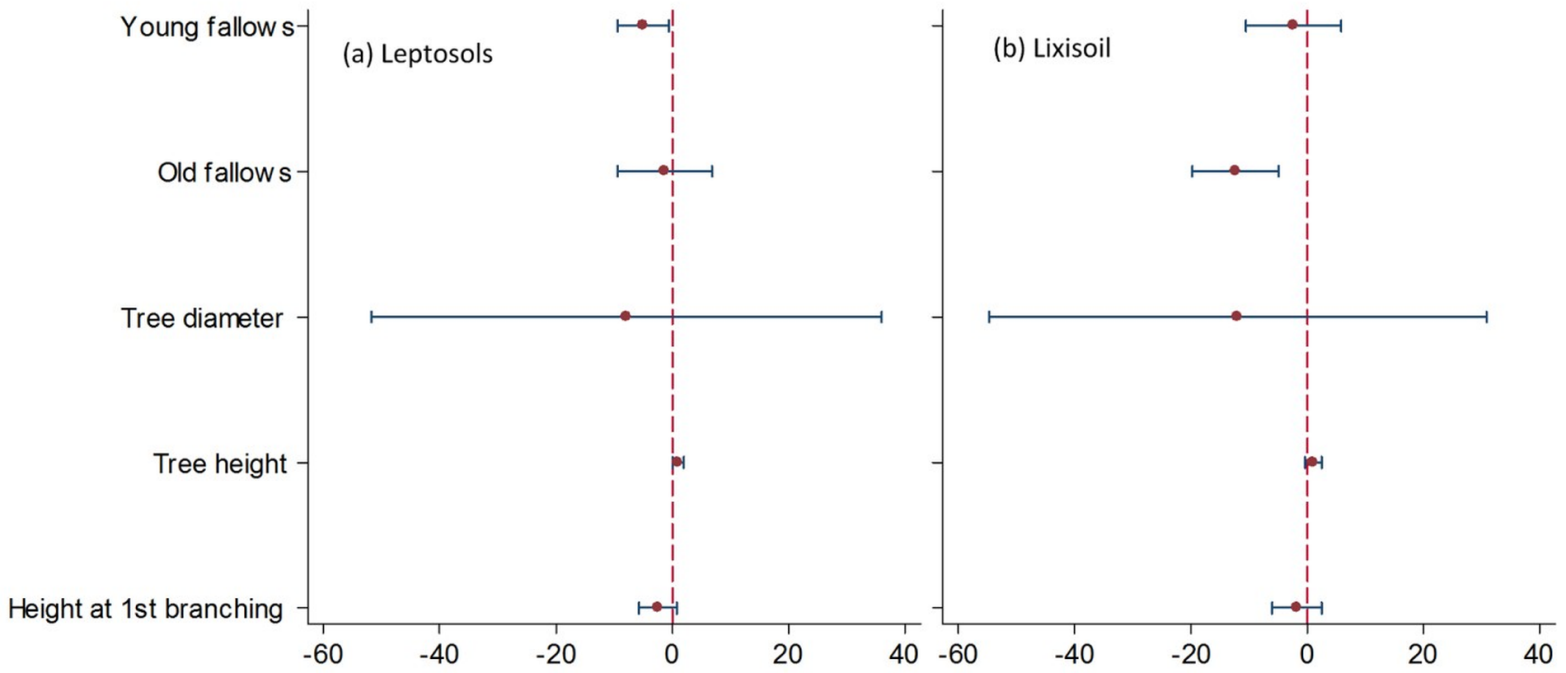
Effects of land-use types and dendrometric parameters on (a) Leptosols (test result for heteroscedasticity was significant: p = 0.0061; *n* = 60; Prob>F = 0.0013; R^2^ = 0.2495) and (b) Lixisols (test result for heteroscedasticity was significant: p = 0.0324;*n* = 60;Prob> F = 0.0230; R^2^ = 0.1587). Variable (y axis) with negative coefficient (x axis) are note associated with fruit yield.

We further explored the specific effect of land-use types with dendrometric parameters for each soil group on shea fruit yield. On leptosols the only dendrometric parameter with a significant effect on fruit yield (Fig 4a) was total height (p = 0.04), which was associated with an increase of nearly one kilogram (b = 0.98) for every one meter increase in total tree height. Among land-use types, young fallow had a significantly negative effect (b = –4.97, p = 0.02) on fruit yield.

Only old fallow plots (b = –12.29, p = 0.00) on lixisols exhibited a significant effect, which was negative, on shea fruit yield (Fig 4b). Tree diameter (b = –11.92, p = 0.57), tree height at first branching (b = –1.72, p = 0.42), tree total height (b = 1.06, p = 0.14), and young fallow (b = –2.34, p = 0.57) had no discernible relationships with fruit yield.

### 3.4 Farm household characteristics

#### 3.4.1. Descriptive statistics

Farm households in the study area managed an average of 5.5 ha (Table 5), although this included land borrowed for crop production by some households, and had a mean size of nine household members, attesting the predominantly subsistence nature of agricultural production. Most respondents (85%) reported using only non-organic fertilizers for soil fertility management, while others reported using either only green and animal manures, or a combination of organic and non-organic fertilizers. A large majority of respondents (93%) conserved shea trees for food, medicine, timber and other uses. In terms of education, more than half of the household heads (55%) were illiterate and 26.5% had attended primary school. Annual gross household income was 935,02 CFA francs (approximately $1,87 USD). The most common means of transportation were bicycles and donkeys. More than half of the respondents belonged to farmers’ organizations.

**Table 5:** Descriptive statistics of farm households in the Atacora study area, Benin (*n* = 183).

Variables*	Mean	Std. Dev.	Min.	Max.
Age of respondent (years)	44	14.5	18	87
Household size (# of members)	9.5	5.3	1	31
Education (dummy variable)	0.7	0.9	0	4
Number of mobile phones	1.3	1.3	0	6
Total landholdings (ha)	5.5	3.1	0.5	18.2
Gross income (CFA franc)	939,433.7	787,693.9	2,700.0	3,514,400
Group membership	0.6	0.7	0	3
Means of transportation (bicycle)	1.2	0.	0	35
Type of fertilizer used (dummy variable)*	1.9	0.6	0	3
Shea trees in farmland (# of trees)	134	94	12	438
Shea trees in fallow areas (# of trees)	52	70	0	376

*Note: Variables selected based on principal component analysis with Kaiser-Meyer-Olkin (KMO) sampling adequacy measure of 0.77

Education level: 0 = illiterate, 1 = Primary, 2 = Secondary and 3 = Tertiary

1$ = 563.88 CFA franc on October 30, 2017

#### 3.4.2. Factors associated with potential fruit yield and land management practices

In addition to morphological and physical site parameters, we examined the relationships between potential fruit yield, socio-economic and management characteristics of farm households. Table 6 summarizes our findings on the association of these factors with the potential fruit yield. Among the socio-economic variables significantly associated with potential fruit yield are total landholding area (b = 2.12, p = 0.00), gross income (b = 0.03; p = 0.00), and distance to the nearest road (b = –0.06; p = 0.00).The number of Shea tree (or density) in fallow areas (b = 0.07; p = 0.00) was the only physical site factor significantly associated with potential fruit yield. Gross income is function of total landholding area managed. The latter determines the contribution of each land-use type and shea tree densities, which in turn affect potential fruit yield. However, greater distance to the nearest road, which is a proxy for the accessibility to transportation facilities and markets, appears to have a direct relationship with potential fruit yield. Households that are closed to the road are more likely to get higher potential yield compared to others. In addition to the regression analysis, we assessed the relative effect of these factors. The greatest effect was exhibited by shea tree density in fallow areas (77%), followed by total landholding size (70%), and the annual gross household income (22%).

**Table 6:** Socio-economic and land management factors associated with total shea fruit yield in the Atacora study area of Benin.

Variable	Coefficient (ß)	Std. Error	95% Confidence Interval	
Distance to nearest road (km)	–0.06	0.0201	–0.10	–0.02
Tree density in fallow areas(# of trees)	0.07	0.002	0.07	0.08
Total landholding area (ha)	2.12	0.13	1.86	2.38
Gross income (CFA franc)	0.03	0.004	0.02	0.04
Borrowed land (ha)	0.18	0.11	–0.03	0.39
Constant	–0.98	0.408	–1.79	–0.18

Note: *n* = 182; R^2^ = 0.94, Prob> F = 0.00

Leaving cultivated areas fallow is a widespread management practice among smallholder farmers in the study area and has an important effect on potential fruit yields (see section 3.2) and the overall condition of shea parklands, therefore we explored factors associated with farmers’ decisions about fallow areas and shea parklands. The relationships between evaluated factors and farmer’s decisions regarding fallow areas are described in Table 7.

**Table 7:** Socio-economic variables associated with households decisions to fallow.

Variables	Coef.	Std. Err.	[95% Conf. Interval]
Education level (dummy variable) *	-1.36	0.53	-2.41–0.31
Land borrowed (ha)	-0.34	0.09	-0.53–0.16
Distance to road (km)	-0.04	0.02	-0.07–0.00
Household size (# of person)	-0.08	0.04	-0.16–0.00
Household phones	0.49	0.19	0.12 0.86
Constant	1.70	0.77	0.18–3.22

Note: n = 178, Pseudo R2 = 0.1553, Prob˃F = 0.00

*Education level: 0 = illiterate, 1 = Primary, 2 = Secondary and 3 = Tertiary

The education level of the household header (b = –1.36, p = 0.01), the area of land borrowed (b = –0.34 p = 0.001), the distance to the road (b = -0.04, p = 0.015) and the size of the household (b = -0.08, p = 0.04) seem to be constraints to fallow practice. The results from Table 7 suggest that, farmers whose education level is above primary level tend to go for other strategies for soil fertility management to the detriment of fallowing. Similarly, the need to feed more people (household size) requires more land for farming and therefore new strategies for soil fertility management leading to the abandonment of fallow practice. Landless farmers or those who borrow lands are reluctant to fallowing probably due to limited availability of lands. The distance from the house to the main road contributed negatively to farmers’ decision to fallow. Nevertheless, it has been noticed that lands that are very far from viability facilities such as roads are the ones given to landless farmers. Again as a result of limited availability of lands, the new tenants cannot afford to leave the given land for fallowing and may probably go for inorganic fertilizer.

The number of phones (b = 0.49, p = 0.009) earned by a given household was the only factors that contributed positively to farmers’ decision to fallow. Though the latter variable has no evident relationship with fallowing, it can be considered as an indicator of wealth which is basically associated to lands ownership. The wealthier the farmer, the higher the area of land owned and the higher the likelihood to practice fallow.

#### 3.4.3. Other related management practices

**Fig 5:**
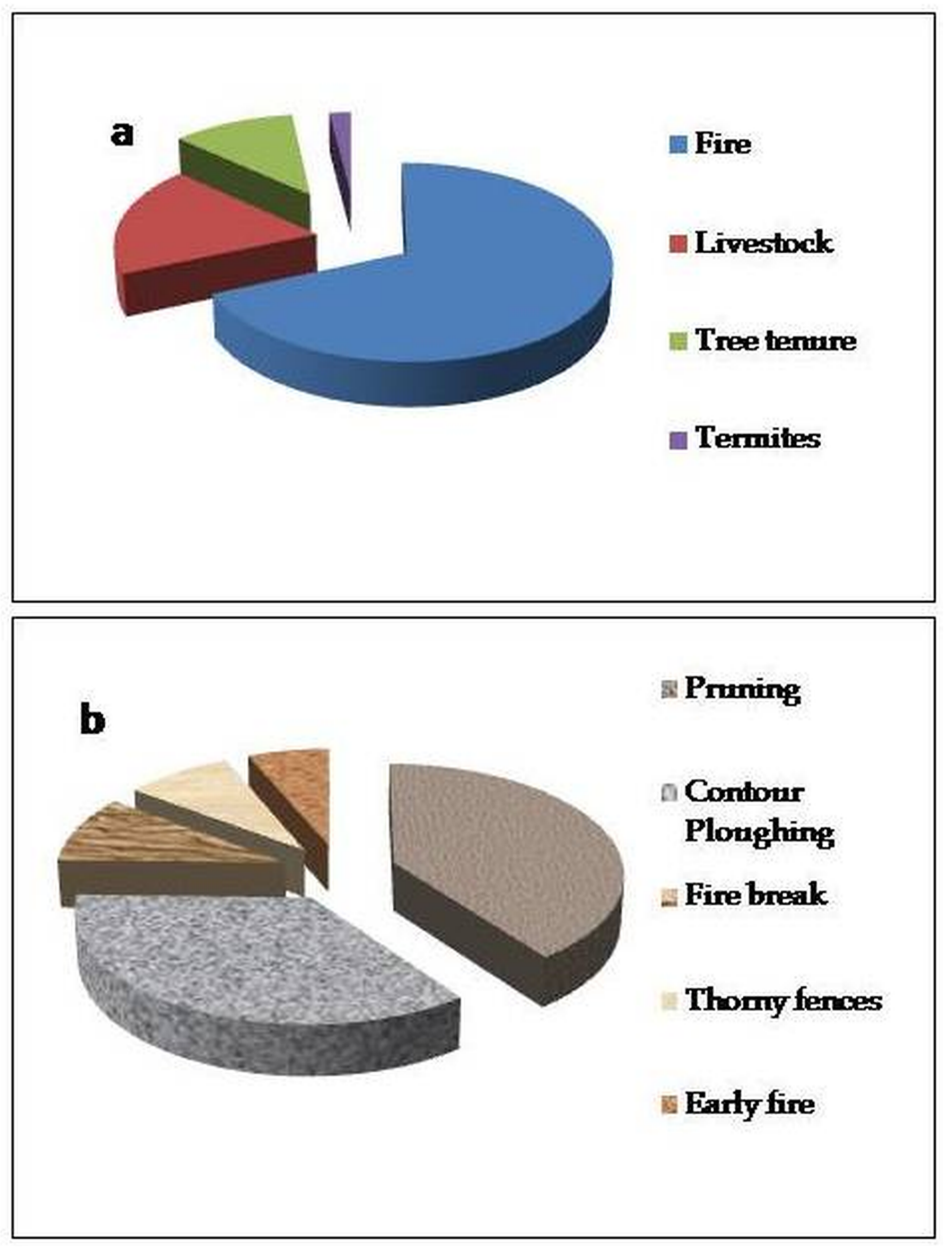
Shea parkland management practices with (a) constraints on shea parkland establishment and (b) shea parkland management practices.

From the household survey data, variables such as fire, livestock grazing, the presences of termites, lack of secure tree tenure, and the lengthy juvenile phase of shea trees were constraints on shea parkland establishment and management (Fig 5a). To address these variables, farmers have developed management strategies to maintain the structure of shea parklands such as fire breaks, contour ploughing, burning early in the season, erecting thorny fences to protect trees from grazing, and pruning (Fig 5b). Fire breaks and contour ploughing are implemented immediately after crops are harvested. Fire breaks are often established around farmland boundaries and/or shea trees. Nearly 22% of survey respondents reported using fire breaks to mitigate the risk of fires in Atacora, whereas 14.2% reported practicing early burning, which lessens the likelihood that fires will damage trees. Early burning is performed from October to mid-November after harvest and before vegetation reaches its driest condition. Due to recent shifts in the timing of cultivation cycles, farmers reported that rather than using the calendar year to determine the burning period, the colour of the vegetation is often used as an indicator for initiating early burning. The use of fences composed of thorny vegetation to protect shea parklands from animal grazing and trampling was reported by a minor proportion (5.6%) of the respondents.

Among the reported management strategies, silvicultural practices such as pruning are used to reduce the crown size near the soil in order to limit shading of crops. Pruning is also used to control infestations of plant parasites. In some cases, shea trees are completely removed; this happens when individuals become old and unproductive.

## 4. Discussion

### 4.1. Effect of land-use, soil group and management practices

The largest *V*. *paradoxa* trees were found in farmland. This may be due to their relatively low and controlled density [9] and the positive impact of low diversity of woody species in farmland. Low density in land under cultivation reduces intra and interspecific competition. Furthermore, soil fertilization from agriculture, originally provided to annual crops, also benefit associated trees. In agroforestry parklands in Nigeria most of the largest *V*. *paradoxa* trees were found in field [37].

Based on the results of our analyses, land use is a determinant factor in shea tree productivity. Farmland registered the highest yield and old fallow as the lowest. Farmland is subject to limits on tree density and diversity as annual crop productivity is the main objective of this land use. Only a few tree species and a limited number of trees were observed on farmland plots. The demographic structure of shea parklands in the study area exhibited an increasing trend in tree density, species diversity, and regeneration along the linear progression from farmland to old fallow [34]. These trends on farmland lead to reduced competition for soil nutrients and water among surviving trees, which in turn may increase the fruit yield of individual trees. Farmland management appears to be an area of potential recommendations for shea parkland productivity.

The inter-annual variability observed for fruit yields in Atacora has been reported by previous studies [4, 38]. This variation might be due to the variability in weather conditions that might affect the tree growth [12] orphenology. Findings of other studies that used similar methods but did not consider land use, estimated yields from 2009 in Bembèrèkè (mean of 251 shea fruit per tree) and Kandi (mean of 305 shea fruit per tree),both located in the Sudanian zone of northern Benin [23], were less than half the yields estimated in this study (means ranging 682 to 963 shea fruit per tree). Nonetheless, the cyclical nature of shea tree productivity is poorly understood[19, 20] and may contribute to the observed inter-annual variability; thus the yield differences may be attributable to 2009 having been a year of poor productivity in the study area. On farmland plots located in the vicinity of the Pendjari Biosphere Reserve in Atacora, a mean of 1,683 shea fruit per tree was recorded in 2011 [24] using the randomized branch sampling method applied in this study.

Apart from the temporal variability of shea fruit yield, the differences between the latter study and this research may be attributable to the sampling method. Akpona et al. [24] applied a method that featured sampling of three main branches of each tree and fruit were counted along each sampled branch, whereas we sampled four main branches and counted fruit on four secondary stems along each main branch sampled. In Burkina Faso, Lamien et al. [39] estimated the mean dry nut to 4 kg per tree accounting for both farmland and fallow land uses. Means ranging between 794 to 1,657 fruit per tree were recorded in shea parklands of Tangrela in northern Cote d’Ivoire [22], which is comparable with production in some results in our study area, but low when compared to the average. This difference may also be due to fact that the Tangrela study covered five consecutive seasons (from 1998 to 2002), which more likely included years of both above and below average shea tree yields. Byakagabaet al.[36] used the randomized branch sampling method of Jessen[35] in Uganda over two consecutive seasons (2009 to 2011) and reported the highest fruit yields on farmland and young fallow plots. In that study productivity was low in the first season with a mean of 193.5 shea fruit per tree on young fallow and 183.3 on farmland plots, while yields in the second season were relatively high with means of 600 and 817 shea fruit per tree for young fallow and farmland plots respectively. The findings of these studies suggest that shea fruit yields are highly variable; however, the limited temporal scale of these studies prevents insight into the long-term temporal patterns of the shea fruit productivity, highlighting the need for long-term research on the subject.

Land-use type is known to be a determinant of shea fruit productivity [24, 39, 40]. Our results suggest that soil should be considered in efforts to optimize productivity. According to the FAO classification, Leptosols are very shallow with many coarse fragments and therefore present limitations to root growth [41]. This group of soil is unattractive for rain fed agriculture because of the limited ability to retain moisture [42]. Lixisols on the other hand have clay enriched subsoil with low activity clays and high base saturation. Unlike Leptosols, Lixisols have some amount of plant nutrient levels with less coarse, making them more suitable for perennial crops. In terms of soil suitability for agricultural planning, Lixisols are more valued than Leptosols.

The strongest association was the combination of Lixisols and farmland, which could be a basis for management research and recommendations. With respect to soil characteristics and fruit yields, Lixisols appear to be better match for shea fruit yield improvement on farmland and young fallow areas, whereas on old fallow, Leptosols are more suitable. Indeed, forestry is the recommended land use on Leptosols according to FAO [43].

### 4.2. Predicting shea fruit yields

The interactions among some dendrometric, soil, and land-use variables were integrated into the shea fruit yield models. We found that management practices, soil type, and tree height had highly significant relationships with shea fruit yield. Lamienet al. [44] developed shea fruit yield prediction models that explained up to 90% of the variation in observed fruit yields using dendrometric parameters and variables such as fruiting density, fruiting intensity and a fruiting index. However, Lamien et al. [44] only considered trees on farmland within a DBH range of 20 to 40 cm over the course of one year. In contrast, we considered widerDBH range (15 to 60 cm) to represent the structure of the shea tree population at the study area over a two year period. These methodological differences might have contributed to the relatively low predictive power of our models. The evaluation of shea fruit yields over a two-year period is a limiting factor in capturing inter-annual variability.

Among the dendrometric parameters used in the prediction model, only total tree height had a significant effect on fruit yield. Similarly, Lamien et al. [44] reported a weak relationship between dendrometric and fruiting variables. Our findings contrast with some previous studies that found positive relationships for tree diameter, crown diameter and crown area and both fruit number and weight [44–46].

There might be other factors that explain shea fruit yield than the ones we considered in this study, which could be perceived as limitation of our study. Furthermore, as found from the survey data, potentially important factors such as fire and winds were not considered in the study design. Late season burning, from December to February, overlaps with shea tree flowering from November to January [47], and are known to reduce fruit yield [3, 38]. Wind occurrence is a challenging issue in Atacora, not only for annual crops but also for perennial trees such as shea. As reported by owners of sampled shea trees, sometime before harvesting, the yield of individual trees can be reduced by half or more by violent winds. In addition to the physical aspects discussed here, intra-specific variability of shea trees might be one of the underlying factors of its fruit productivity [48].

### 4.3. Effect of household characteristics on shea parklands management and conservation

The analysis of household socioeconomic factors is complementary to analysis of land use, soil type and management practices (previous section).The results of both analyses underscored the relationships between fallow land (i.e., old fallow) and productivity. Since the objective of farmers is to improve yields, soil groups and management practices should be appropriately considered; whereas some household socio-economic characteristics (e.g., distance to nearest road, gross annual household income and land tenure) influenced farmers’ decisions regarding management practices.

Ruthenberg [49] and [50] traced the process of intensification of tropical farming systems, resulting from increasing population pressure on the land, as passing from shifting systems through fallow systems to permanent systems. Similarly, increasing land shortages concomitant with rising population densities are resulting in an increase in the production of permanently cultivated fields[51] in [52].

The potential benefit from shea parklands in terms of primary production and smallholder farmers livelihoods depend on management[53]. Therefore understanding management is crucial for the improvement of shea tree resources [10, 27, 46, 54–56]. For example, partial pruning (as reported by survey respondents in this study) and total pruning of shea trees have proved to be successful means of rejuvenating productivity of older trees 5 to 6 years after pruning [57, 58]. For policy recommendation considerations, this finding opens up new possibilities to assist smallholder farmers in the study area and other shea producing regions in Africa. Okiror et al. [27] and Okullo et al. [47] reported that a lack of tree tenure was a limiting factor to shea tree establishment in Uganda. Also distance to viability facilities influences farmers decisions towards parklands management [59]. *V*. *paradoxa* is a slow growing species, requiring 15 to 20 years before initial flowering [60, 61] and reaching maximum productivity at around 50 years of age[17]. The delayed maturity of shea trees tends to discourage farmers from investing in shea tree plantations and makes natural regeneration and passive management more cost effective. Attempts have been made to reduce the juvenile phase of shea trees through the use of nurseries, grafting and improved nutrient supply [11, 17, 62] with encouraging outcomes that are yet to be disseminated. Meanwhile farmers are attracted to other trees such as *Anacardiumoccidentale* L., *Eucalyptus* spp. and *Tectonagrandis* L.f. for plantation projects[63].

## 5. Conclusions

*Vitellaria paradoxa* parklands are one of the dominant features of the Sudan savannah. The species plays a very important role in the economic and social life of smallholder farmers in SSA. Currently, there is a renewed interest in protecting and improving shea resources as domestic and international demand for shea kernel and shea butter increases. This attempt to characterize shea tree fruit yield in Atacora District was successful in the sense that observed fruit yields were comparable to records from other shea producing areas. Findings in northern Benin provide knowledge on the considerable potential for yield improvement through adequate management practices. Soil groups and management practices should be considered for optimizing shea trees yields, whereas certain household socio-economic characteristics (e.g., distance to nearest road, gross annual household income and land tenure) were found to influence farmers’ decisions regarding management practice choices. Our findings emphasize the cyclical nature of shea productivity, but the long-term patterns continue to be poorly understood. The erratic nature that characterizes fruit yield implies that there is room for improvement provided adequate management is applied. Interestingly, land-use and soil group coupled with dendrometric parameters and household characteristics governing management strategies can be used to predict the total yield of shea parklands. Long-term research efforts of the described phenomenon are needed to determine the patterns of shea productivity and to improve the potential for using physical and morphological parameters for predicting shea yield. The reluctance among farmers to plant shea trees, owned not only to its lengthy juvenile phase, but also to taboos against planting shea trees and to the difficulty in growing them due to the recalcitrant seed. This emphasizes the need for dissemination of improved materials and adequate management practices.

## Supporting information

S1 File Shea fruit productivity assessment in Atacora department, Benin. (DOCX)

S2 File Questionnqire sheet. (DOCX)

S1 Table Data collection sheet. (DOCX)

S2 Table Shea fruit yield in Atacora. (DOCX)
