# Supplementary material for: Shea (Vitellaria paradoxa Gaertn C. F.) fruit yield assessment and management by farm households in the Atacora district of Benin
Source: PLoS One. 2018 Jan 18;13(1):e0190234. doi: 10.1371/journal.pone.0190234 (PMC5773006; doi:10.1371/journal.pone.0190234)
Supplement: S1 File — (DOCX) [file pone.0190234.s001.docx]

**S1 F. Shea fruit Productivity assessment in Atacora department, Benin**

Shea fruits annual productivity was evaluated in Atacora district based on 2 treatments.

- The first set concern the soil; 5 groups of soil occurred in the study area: (a) Lixisols, (b) Leptosols (c) Fluvisols, (d) Nitisols and (e) Plinthosols. The 3 most represented soil units were considered. These are the first 3 ones on the list namely: (a) Lixisols, (b) Leptosols and (c) Fluvisols.

During the field work, Fluvisols were discarded from the analysis due the absence of shea trees on that type of soil.

- The second set refers to the land use; three land use precisely farmland, young fallow (from one to five years of fallowing) and old fallow (more than 5 years of fallowing) were considered.

On each soil map unit, six (06) plots (50 m × 50 m) were established in farmland, six (06) in young fallow and three (03) in old fallows. Within each plot four (04) fruiting shea trees were randomly selected for yield evaluation. A total of 60 fruiting shea trees (*n* = 24 for farmland, *n* = 24 for young fallow and *n* = 12 for old fallow) were monitored on each of the two soil group for two consecutive growing seasons: from 2013 to 2015. All the one hundred and twenty (120) trees monitored were labeled with a metallic plaque.

Fruit yield was measured following the Randomized Branch Sampling Method. It consists of sampling branches from the first branching of the stem. The process is summed up as follow:

1. Count the number of main branches on each selected tree,
2. Select four (04) main branches from the total number of branches (number of branches recorded in step 1) for fruit yield evaluation,
3. Count the number of branching along the path of each selected main branch,
4. Select randomly four (04) secondary branches along the path of each main branch,
5. Follow each selected secondary branch to the terminal segment and count fruits carefully.
6. Count the number of fruit along each selected secondary branch up to the terminal segment,

Fruit yield in terms of number of fruit for each selected main branch will be derived from the pooled number of fruits counted on the four (04) secondary branches and the number of branching along the path of the main branch (recorded in step 5).

The total fruit yield of a tree will be estimated from the average fruit yield of a main branch and the total number of main branches of each tree (number recorded in step 1).

**Fruits weight evaluation**

Select ten (10) fruits on each tree for fruit weight estimation, label each sample, and follow step *a* to *d*.

1. Weight ten (10) fresh fruit with the pulp (W1),
2. Remove the pulp and weight the fruit again, this time without pulp (W2),
3. Dry the fruit under the sun and continue weighting until the weight remain stable,
4. Weight the dried fruit again (W 4).

Shea tree productivity in terms of fruit weight (fresh and dry weight) will be derived from the calculated yield (number of fruit bore by a tree) and the weight obtains from 10 fruits.
