# Supplementary material for: Shea (Vitellaria paradoxa Gaertn C. F.) fruit yield assessment and management by farm households in the Atacora district of Benin
Source: PLoS One. 2018 Jan 18;13(1):e0190234. doi: 10.1371/journal.pone.0190234 (PMC5773006; doi:10.1371/journal.pone.0190234)
Supplement: S2 File — (DOCX) [file pone.0190234.s002.docx]

**S2 File. Questionnaire sheet**

N° … Date:

Uses and management of Shea parklands in northern Benin

**Statement of intent**

The purpose of this questionnaire is basically to learn from stakeholders engaged in Shea parklands management. The information obtained from this study will under no circumstances be used to evaluate any respondent. It is to be used by the researcher in writing thesis research project to fulfil the requirement of an award of a PhD degree in the area of climate change and agriculture.

1. **Identification of the interviewee**

I.1 Name: Sex: Matrimonial situation:

I.2 Commune: Village: locality:

I.3 Household Coordinates X:_____________: Y:______________

1. **Characterization of the household**

II.1 Size of the household

II.2 Quality of the household

II.3 Age of the household header

II.4 Who is the head of the household?

II.5 Matrimonial status of the household header

II.6 Profession of the head of household

1. Farmer: b. Breeder: c. Housewife: d. Trader: e. No profession: f. Other (specify):

II.7 Language of discussion:

II.8 Ethnic group: Husband: Wife 1: Wife 2:

II.9 Income per month (in CFA):

a. ≤ 31 625 (specify if possible) b. 31 625 to 45 000 c. 45 000 to 60 000 d. ≥ 60 000 (specify if possible)

What is the source of the income given in 9

II.10 Level of instruction

a. Illiterate: b. Primary: c. J H S:

d. Senior High School: e. University: f. Others:

II.10 Do you use a phone?

II.11 If yes, how many phones does your household own?

II.12 Are you a member of cooperatives or organized groups?

II.13 If yes how many

II.14 What are they?

II.15 Household’s wealth (indicate the number of each item)

Radio TV Fan DVD/CD Car Bicycle Motorbike Cow Sheep Goat Pig Poultry

Others:

II.16 What is the distance from your house to the main road?

1. **Farming system**

III.1. what is the total surface of your lands?

III.1. a. <1 ha III.1. b. 1-5 ha III.1. c. 5-10 ha III.1. d. > 10 ha

III.2 What is the number of plots?

**III.3 Land ownership**

| Type of land use | Owned and cultivated | | Owned but cultivated by others | | Owned but not cultivated | | Cultivated but owned by others | | Total | |
| --- | --- | --- | --- | --- | --- | --- | --- | --- | --- | --- |
|  | Total plots | Area (ha) | Total plots | Area (ha) | Total plots | Area (ha) | Total plots | Area (ha) | Total plots | Area (ha) |
| 1. Corn field |  |  |  |  |  |  |  |  |  |  |
| 2. Sorghum field |  |  |  |  |  |  |  |  |  |  |
| 3. Peanut field |  |  |  |  |  |  |  |  |  |  |
| 4. Cow pea field |  |  |  |  |  |  |  |  |  |  |
| 5. Cotton |  |  |  |  |  |  |  |  |  |  |
| 6. Corn + Sorghum |  |  |  |  |  |  |  |  |  |  |
| 7. Sorghum + Peanut |  |  |  |  |  |  |  |  |  |  |
| 8. Cow pea + sorghum |  |  |  |  |  |  |  |  |  |  |
| 9. Cotton + Cow pea |  |  |  |  |  |  |  |  |  |  |
| 10. Fonio |  |  |  |  |  |  |  |  |  |  |
| Perennial crop field |  |  |  |  |  |  |  |  |  |  |

**III.4**. Physical characteristics of agricultural land

| Type | Distance from the house to the field | Slope of the area  *1= flat*  *2=slight*  *3= moderate*  *4= steep* | Top soil colour & texture  *Colour: Texture:*  *1- black a- sandy 2- yellow b- silty*  *3- red c- loamy*  *4- light d- clayey*  *5-others* | Soil Fertility  *1=poor; 2=moderate;*  *3=good* | Soil fertility in past 5 years  *1=decreasing; 2=stable; 3=increasing* | Fertilizer application  Do you apply fertilizer?  (*0= No;*  *1=green manure;*  *2= animal manure;*  *3 = Inorganic, e.g. NPK)* | Planting frequency  How many times do you plant a year? |
| --- | --- | --- | --- | --- | --- | --- | --- |
| **1** |  |  |  |  |  |  |  |
| **2** |  |  |  |  |  |  |  |
| **3** |  |  |  |  |  |  |  |
| **4** |  |  |  |  |  |  |  |
| **5** |  |  |  |  |  |  |  |
| **6** |  |  |  |  |  |  |  |
| **7** |  |  |  |  |  |  |  |
| **8** |  |  |  |  |  |  |  |
| **9** |  |  |  |  |  |  |  |
| **10** |  |  |  |  |  |  |  |

1, 2, …10 are respectively equivalent to crops cited in III.3

**III.5** Land tenure system

| **Mode of land acquisition**  1- inheritance  2- newly & freely cleared  3- use rights given by local leaders  4- Benefits sharing  5- Buying  6- Rental  7- others | How are land rights secured?  0- none  1- crops /trees on it  2 -sale receipt  3- customary regulation  4- others | Can land be inherited by children male and female? (Y/N)  If yes, what is the plan if children got married) | Land title (Y/N) | Land right | | |
| --- | --- | --- | --- | --- | --- | --- |
|  |  |  |  | Rent | Share crop | Pawn  Sell |

III.6 What is the yield of different crop of the recent agricultural period? (Precise the surface cropped in ha and the yield in tones, Kg, bags or number of bowl).

**1- 2- 3-**

**4- 5- 6-**

**Soil fertility management**

III.7 Do you observe fallow? Y/N

III.8 If no, why?

III.9 If yes why do you practice fallow? (multiple answers are possible)

a- Low crop yield b- Lack of labour

c- Expect to see forest again d- For soil recovering

e- Too far from home f- Other reasons:

If more than 2 choices, ask farmer to rank their reasons according to importance:

| Rank | Reasons for fallow |
| --- | --- |
| 1 |  |
| 2 |  |
| 3 |  |
| 4 |  |
| 5 |  |

**III.10** For what do you use your land before leaving it to fallow?

a- Annual or short cycle crops for how long? _______ Yrs.

b- Perennial crops for how long? _______ Yrs.

c- Pasture for how long? _______ Yrs.

d- Other use for how long? _______ Yrs.

**III.11** How long do you need to leave the plot to reach its higher productivity? ________ Years

**III.12** How do you know when a fallow can be cropped again for good yield?

1. Vegetation characteristics (e.g. type/species/ tree density/ tree size/ etc.)
2. Soil characteristics (colour of the top soil/ soil depth/soil organisms/ soil moisture, etc)
3. Fallow length: _________ years
4. **General information on Shea tree**

IV.1 Do you know any tree called Shea or Karite tree?

IV.2 How do you call it in your dialect?

IV.3 Is the tree useful to you? Y/N

IV.4 What is it used for?

1. Food: which part of the plant ?
2. Medicine: which part of the plant ?
3. Fuel Wood: which part of the plant ?

d. Cosmetic: which part of the plant ?

e. Construction: which part of the plant ?

f. Others (specify):

IV.5 Which part(s) of the plant cited above are more important for you?

| Utile Parts? | Importance | observations | Utile Parts? | Importance | observations |
| --- | --- | --- | --- | --- | --- |
| Pulp |  |  | Roots |  |  |
| Kernel |  |  | Wood |  |  |
| Bark |  |  | Leaves |  |  |
|  |  |  |  |  |  |

1. **Establishment, role and Management strategies of Shea parklands**

V.1 Do you have Shea tree in your field? Y/N

V.2 If yes how is it established?

a. Conservation: b. Plantation: c. Others (specify):

V.3 Which of these is your preference

V.3’ Why this preference?

V.4 Do you encounter problems in the establishment of Shea trees? Y: N:

V.5 What kinds of problems do you encounter when establishing Shea tree?

**V.5.a** Animals **V.5.b** Unts

**V.5.c** Pests and diseases **V.5.d B**ush fire

**V.5.e** Others (specify)

V.6 How do you overcome these Problems?

V.7 Could you state the importance of Shea tree in your field?

**V.7.a** Food for people Yes: No:

**V.7.b** Fodder for animals Yes: No:

**V.7.c** Wood for household Yes: No:

**V.7.d** wind and water breaks Yes : No:

**V.7.e** Shade for people and animals Yes: No:

**V.7.f** Medicine purpose Yes: No:

**V.7.g** Soil fertility management Yes: No:

**V.7.h** Other …………………..

V.8 Do Shea trees in your fields have any influence on crops? Yes: No:

V.9 If yes which crops

V.10 How do Shea trees influence the crop?

V.11 How do you manage it?

V.12 If the influence is negative on the crops why do you still grow them?

V.13 Is there any law protecting Shea trees in this area? Yes; No;

V.14 And you, do you protect shea trees in your field? Yes; No

V.15 If yes how is the protection done?

1. Protection against bushfire,
2. Protection against livestock during juvenile phase
3. Conservation of juvenile during tillage and thinning,
4. Never cut, but pruned
5. Contour ploughing
6. Others

V.16 If not, why?

1. **Social organization and collection of Shea nut**

VI.1 Do you collect Shea fruit?

VI.2 Where do you collect Shea nut?

a. Fields b. Fallows c. Forest

VI.3 How far do you go usually to collect Shea nut?

1. Not far b. Close house c. Far d. Very far

VI.4 Do you easily have access to Shea Nuts? Yes No

VI.4 If no, why?

VI.5 Who owns Shea nut in your locality?

1. The person who collect it b. Landlord

c. The current tenant d. Everybody

e. Others (specify)

VI.6 How is Shea nut collection done?

1. Individually b. With family members c. In groups

VI.7 Which kind of people in your locality use to collect Shea nuts?

1. Male: b. female:

d. Youths: e. Adults: f. Old:

VI.8 For how long do you usually collect Shea nuts in a year?

VI.8.a. One month: VI.8.b. Two months: VI.8.c. Three months: VI.8.d. More:

VI.9 Which period of the year is this activity held?

VI.10 How much nuts can you as household collect per year?

VI.11 What is the use of the Shea nuts collected?

1. Pulp: a_1_. Food Yes / No a_2_. Medicine Yes / No

a_3_. Cosmetic Yes / No a_4_. Marketing Yes / No

1. Kernel:

a_1_. Food Yes / No b_2_. Medicine Yes/No

c_3_. Cosmetic Yes/No a_4_. Marketing Yes / No

1. **Process of Shea butter manufacture**

VII.1 How are Shea nuts processed to butter?

1. Manually Y/N b. Mechanically Y/N

VII.2 What is the use of the processed Shea butter?

1. Household consumption: Y/N or/ b. Commercial Purpose:

VII.3 Do you always sell Shea nut and butter? Y/N

VII.4 If no, when or under which condition do you proceed to the commercialization of Shea butter?

VII.5 To whom in the household belongs income from Shea products a The husband b. the wife c. both d. Anyone who collect it

VII.6 Does the income from Shea products have an impact in your livelihood? Yes No

VII.7 If yes, How?

1. Reducing food insecurity
2. Increasing household income
3. Others …….
