# Supplementary material for: Shea (Vitellaria paradoxa Gaertn C. F.) fruit yield assessment and management by farm households in the Atacora district of Benin
Source: PLoS One. 2018 Jan 18;13(1):e0190234. doi: 10.1371/journal.pone.0190234 (PMC5773006; doi:10.1371/journal.pone.0190234)
Supplement: S1 Table — (DOCX) [file pone.0190234.s003.docx]

**S1 Table. Data collection sheet**

Date: Plot N°:

Soil type: Land use type: Tree N°:

Commune: Village: Locality:

Latitude: Longitude: Altitude:

| Number of branches | Tree’s Circumference | Total height | Height at first branching | Crown diameter | |
| --- | --- | --- | --- | --- | --- |
|  |  |  |  | N-S | E-W |
|  |  |  |  |  |  |

| Branch N° | Secondary branch | Number of fruits |  | Branch N° | Secondary branch | Number of fruits |
| --- | --- | --- | --- | --- | --- | --- |
| B I | B I-1 |  |  | B II | B II-1 |  |
| Number of branching on B I | B I-2 |  |  | Number of branching on B I | B II-2 |  |
|  | B I-3 |  |  |  | B II-3 |  |
|  | B I-4 |  |  |  | B II-4 |  |
|  | | | | | | |
| Branch N° | Secondary branch | Number of fruits |  | Branch N° | Secondary branch | Number of fruits |
| B III | B III-1 |  |  | B IV | B IV-1 |  |
| Number of branching on B I | B III-2 |  |  | Number of branching on B IV | B IV-2 |  |
|  | B III-3 |  |  |  | B IV-3 |  |
|  | B III-4 |  |  |  | B IV-4 |  |

Note: BI, BII, BIII and BIV refers to the four main branches selected for fruit counting

BI-1 to BI-4 refers to the four secondary branches selected on the main branch I and so on.

N-S refers to North South direction; E-W refers to East West direction.
