# Supplementary material for: Shea (Vitellaria paradoxa Gaertn C. F.) fruit yield assessment and management by farm households in the Atacora district of Benin
Source: PLoS One. 2018 Jan 18;13(1):e0190234. doi: 10.1371/journal.pone.0190234 (PMC5773006; doi:10.1371/journal.pone.0190234)
Supplement: S2 Table — (DOCX) [file pone.0190234.s004.docx]

**S2 Table. Shea fruit yield in Atacora**

| **Land use type** | **Tree N°** | **Soil type** | **Productivity -2014** | **Productivity-2015** | **Mean Productivity**  **(number of fruits)** | **Fruit weight (in kg)** |
| --- | --- | --- | --- | --- | --- | --- |
| Field | 1 | 1 | 979,8 | 235,3 | 607,5 | 6,8 |
| Field | 2 | 1 | 2008,2 | 563,1 | 1285,6 | 30,3 |
| Field | 3 | 1 | 832,1 | 57,0 | 444,6 | 11,1 |
| Field | 4 | 1 | 2911,9 | 229,9 | 1570,9 | 18,9 |
| Field | 5 | 1 | 1242,5 | 1598,1 | 1420,3 | 31,1 |
| Field | 6 | 1 | 213,8 | 2177,3 | 1195,6 | 14,9 |
| Field | 7 | 1 | 1315,6 | 2375,0 | 1845,3 | 30,6 |
| Field | 8 | 1 | 186,5 | 186,5 | 186,5 | 2,4 |
| Field | 9 | 1 | 2126,4 | 2162,2 | 2144,3 | 27,9 |
| Field | 10 | 1 | 5929,5 | 2550,0 | 4239,8 | 64,0 |
| Field | 11 | 1 | 783,8 | 1632,2 | 1208,0 | 24,2 |
| Field | 12 | 1 | 1114,3 | 412,3 | 763,3 | 14,1 |
| Field | 13 | 1 | 725,4 | 588,0 | 656,7 | 11,9 |
| Field | 14 | 1 | 1527,0 | 591,0 | 1059,0 | 20,2 |
| Field | 15 | 1 | 126,0 | 510,0 | 318,0 | 7,3 |
| Field | 16 | 1 | 817,9 | 817,9 | 817,9 | 8,3 |
| Field | 27 | 1 | 643,8 | 292,1 | 467,9 | 4,9 |
| Field | 28 | 1 | 2206,0 | 0,0 | 1103,0 | 23,7 |
| Field | 29 | 1 | 883,8 | 1585,5 | 1234,6 | 27,8 |
| Field | 30 | 1 | 682,5 | 322,6 | 502,5 | 8,9 |
| Field | 45 | 1 | 841,5 | 1391,9 | 1116,7 | 15,6 |
| Field | 46 | 1 | 1624,4 | 1253,5 | 1438,9 | 22,4 |
| Field | 47 | 1 | 1024,0 | 120,0 | 572,0 | 21,2 |
| Field | 48 | 1 | 1104,0 | 511,0 | 807,5 | 19,1 |
| Field | 81 | 2 | 441,0 | 378,0 | 409,5 | 5,7 |
| Field | 82 | 2 | 1433,3 | 926,6 | 1179,9 | 23,7 |
| Field | 83 | 2 | 598,0 | 1303,3 | 950,6 | 17,4 |
| Field | 84 | 2 | 209,7 | 613,3 | 411,5 | 10,3 |
| Field | 85 | 2 | 1591,6 | 921,2 | 1256,4 | 17,8 |
| Field | 86 | 2 | 86,0 | 277,5 | 181,8 | 2,6 |
| Field | 87 | 2 | 1568,0 | 37,5 | 802,8 | 10,3 |
| Field | 88 | 2 | 286,1 | 0,0 | 143,1 | 3,8 |
| Field | 89 | 2 | 1396,7 | 0,0 | 698,3 | 14,5 |
| Field | 90 | 2 | 309,8 | 171,9 | 240,8 | 4,0 |
| Field | 91 | 2 | 760,0 | 356,9 | 558,4 | 17,0 |
| Field | 92 | 2 | 873,7 | 0,0 | 436,8 | 6,6 |
| Field | 93 | 2 | 1495,5 | 1495,5 | 1495,5 | 31,4 |
| Field | 94 | 2 | 183,9 | 374,1 | 279,0 | 3,5 |
| Field | 95 | 2 | 1839,4 | 600,0 | 1219,7 | 24,9 |
| Field | 96 | 2 | 1767,5 | 347,4 | 1057,4 | 28,1 |
| Field | 97 | 2 | 1035,0 | 771,3 | 903,1 | 20,0 |
| Field | 98 | 2 | 1743,6 | 1215,5 | 1479,6 | 27,7 |
| Field | 99 | 2 | 224,8 | 366,4 | 295,6 | 8,2 |
| Field | 100 | 2 | 122,4 | 0,0 | 61,2 | 0,9 |
| Field | 101 | 2 | 822,9 | 2720,8 | 1771,9 | 34,6 |
| Field | 102 | 2 | 1418,4 | 1739,1 | 1578,8 | 26,5 |
| Field | 103 | 2 | 1599,0 | 540,0 | 1069,5 | 21,0 |
| Field | 104 | 2 | 869,3 | 647,2 | 758,2 | 8,6 |
| Young fallow | 17 | 1 | 376,1 | 898,9 | 637,5 | 17,9 |
| Young fallow | 18 | 1 | 106,5 | 0,0 | 53,3 | 1,3 |
| Young fallow | 19 | 1 | 445,5 | 0,0 | 222,8 | 4,9 |
| Young fallow | 20 | 1 | 2223,0 | 0,0 | 1111,5 | 28,5 |
| Young fallow | 21 | 1 | 279,6 | 182,3 | 230,9 | 3,9 |
| Young fallow | 22 | 1 | 934,7 | 0,0 | 467,3 | 5,6 |
| Young fallow | 23 | 1 | 95,6 | 0,0 | 47,8 | 0,7 |
| Young fallow | 24 | 1 | 169,8 | 936,3 | 553,0 | 14,5 |
| Young fallow | 25 | 1 | 1014,8 | 0,0 | 507,4 | 8,4 |
| Young fallow | 26 | 1 | 379,5 | 2176,5 | 1278,0 | 12,4 |
| Young fallow | 31 | 1 | 2581,8 | 2232,4 | 2407,1 | 51,3 |
| Young fallow  Young fallow | 32 | 1 | 2048,8 | 1727,0 | 1887,9 | 43,2 |
|  | 33 | 1 | 549,4 | 0,0 | 274,7 | 3,7 |
| Young fallow | 34 | 1 | 617,3 | 2406,6 | 1511,9 | 30,8 |
| Young fallow | 35 | 1 | 686,6 | 292,5 | 489,5 | 9,8 |
| Young fallow | 36 | 1 | 188,8 | 188,8 | 188,8 | 3,1 |
| Young fallow | 37 | 1 | 581,2 | 0,0 | 290,6 | 4,8 |
| Young fallow | 38 | 1 | 1003,0 | 0,0 | 501,5 | 7,7 |
| Young fallow | 39 | 1 | 345,0 | 0,0 | 172,5 | 2,9 |
| Young fallow | 40 | 1 | 687,0 | 3093,0 | 1890,0 | 25,1 |
| Young fallow | 41 | 1 | 1129,6 | 1529,5 | 1329,6 | 33,0 |
| Young fallow | 42 | 1 | 1436,3 | 2502,0 | 1969,1 | 42,1 |
| Young fallow | 43 | 1 | 412,5 | 979,4 | 695,9 | 16,9 |
| Young fallow | 44 | 1 | 332,8 | 1697,8 | 1015,3 | 20,9 |
| Young fallow | 73 | 2 | 449,1 | 119,1 | 284,1 | 4,6 |
| Young fallow | 74 | 2 | 321,6 | 526,9 | 424,2 | 10,4 |
| Young fallow | 75 | 2 | 171,9 | 229,6 | 200,8 | 4,7 |
| Young fallow | 76 | 2 | 646,3 | 546,8 | 596,5 | 9,2 |
| Young fallow | 77 | 2 | 1265,0 | 175,4 | 720,2 | 12,0 |
| Young fallow | 78 | 2 | 296,5 | 86,5 | 191,5 | 5,2 |
| Young fallow | 79 | 2 | 95,8 | 376,3 | 236,0 | 4,2 |
| Young fallow | 80 | 2 | 506,6 | 1651,1 | 1078,9 | 21,4 |
| Young fallow | 105 | 2 | 1063,5 | 1035,8 | 1049,6 | 13,9 |
| Young fallow | 106 | 2 | 490,3 | 460,3 | 475,3 | 6,5 |
| Young fallow | 107 | 2 | 530,3 | 2510,8 | 1520,5 | 11,4 |
| Young fallow | 108 | 2 | 727,5 | 1095,0 | 911,3 | 14,3 |
| Young fallow | 109 | 2 | 485,7 | 1176,8 | 831,3 | 18,5 |
| Young fallow | 110 | 2 | 195,3 | 0,0 | 97,7 | 1,2 |
| Young fallow | 111 | 2 | 369,0 | 102,0 | 235,5 | 4,0 |
| Young fallow | 112 | 2 | 320,5 | 819,0 | 569,8 | 10,7 |
| Young fallow | 113 | 2 | 1779,4 | 1345,0 | 1562,2 | 22,2 |
| Young fallow | 114 | 2 | 884,8 | 166,5 | 525,7 | 9,5 |
| Young fallow | 115 | 2 | 175,9 | 97,6 | 136,7 | 3,5 |
| Young fallow | 116 | 2 | 26,5 | 203,5 | 115,0 | 1,0 |
| Young fallow | 117 | 2 | 747,0 | 324,0 | 535,5 | 7,0 |
| Young fallow | 118 | 2 | 393,9 | 0,0 | 197,0 | 3,0 |
| Young fallow | 119 | 2 | 477,5 | 436,3 | 456,9 | 5,8 |
| Young fallow | 120 | 2 | 115,9 | 0,0 | 58,0 | 0,7 |
| Old fallow | 49 | 1 | 546,8 | 546,8 | 546,8 | 7,4 |
| Old fallow | 50 | 1 | 84,0 | 84,0 | 84,0 | 1,5 |
| Old fallow | 51 | 1 | 45,5 | 45,5 | 45,5 | 0,6 |
| Old fallow | 52 | 1 | 783,3 | 783,3 | 783,3 | 5,5 |
| Old fallow | 53 | 1 | 766,5 | 951,1 | 858,8 | 18,2 |
| Old fallow | 54 | 1 | 503,9 | 710,2 | 607,1 | 9,7 |
| Old fallow | 55 | 1 | 808,5 | 1693,3 | 1250,9 | 25,9 |
| Old fallow | 56 | 1 | 500,5 | 464,8 | 482,6 | 12,3 |
| Old fallow | 57 | 1 | 75,3 | 0,0 | 37,6 | 0,5 |
| Old fallow | 58 | 1 | 92,5 | 377,5 | 235,0 | 2,8 |
| Old fallow | 59 | 1 | 301,5 | 175,5 | 238,5 | 2,4 |
| Old fallow | 60 | 1 | 139,0 | 0,0 | 69,5 | 1,0 |
| Old fallow | 61 | 2 | 2541,4 | 1667,3 | 2104,4 | 22,7 |
| Old fallow | 62 | 2 | 785,0 | 1703,8 | 1244,4 | 19,2 |
| Old fallow | 63 | 2 | 886,9 | 327,2 | 607,0 | 5,9 |
| Old fallow | 64 | 2 | 612,5 | 1648,5 | 1130,5 | 15,5 |
| Old fallow | 65 | 2 | 1037,8 | 1915,3 | 1476,6 | 36,9 |
| Old fallow | 66 | 2 | 219,3 | 1614,9 | 917,1 | 12,0 |
| Old fallow | 67 | 2 | 521,5 | 1324,8 | 923,1 | 14,9 |
| Old fallow | 68 | 2 | 808,5 | 358,5 | 583,5 | 5,7 |
| Old fallow | 69 | 2 | 645,8 | 508,4 | 577,1 | 4,2 |
| Old fallow | 70 | 2 | 1210,6 | 4606,9 | 2908,8 | 39,8 |
| Old fallow | 71 | 2 | 462,2 | 478,1 | 470,2 | 6,8 |
| Old fallow | 72 | 2 | 501,0 | 0,0 | 250,5 | 3,3 |

Soil type : 1= Lixisols, 2=Leptosols
